# Supplementary material for: Molecular Basis of Müllerian Agenesis Causing Congenital Uterine Factor Infertility—A Systematic Review
Source: Int J Mol Sci. 2023 Dec 21;25(1):120. doi: 10.3390/ijms25010120 (PMC10778982; doi:10.3390/ijms25010120)
Supplement: Supplementary file 1 [file ijms-25-00120-s001.zip › ijms-2695126-supplementary.pdf]

1. STUDIES INCLUDED

	Author/Year [Reference]	Type of Study	Population/ Sample	Findings	Additional Findings
1.	Thomson, E, 2023 [42]	Case series	13	-CNV- 1.4Mb deletion at 17q12 in proband MRKHS. Contains the candidate genes *LHX1* and *HNF1B*.<br>- Other CNVs- duplication at 3q13. 12q13.13 in proband MRKHS contains three main genes—IFTP57, HHLA2 and MYH15<br>- Microdeletion at Xp22.33 within the upstream conserved non-coding elements enhancer region of SHOX gene<br>- duplication at 2q24.2 of proband MRKHS involved *PLA2R1*, *ITGB6* and *RBMS1*	SNP microarray analysis<br>Included the in-vivo study in mice
2.	Ragitha, TS, 2023 [76]	Cohort	32 with MA and/or gonadal dysgenesis	Single nucleotide variations in WNT4 gene (n=3). Synonymous polymorphism showed the absence of microRNA regulatory sites (n=1). A nucleotide substitution in intronic regions that did not affect the normal splicing mechanism (n=2)	Also included those with gonadal dysgenesis
3.	Brakta, S, 2023 [98]	Case-control	87 with MRKHS and available parents	14 SVs were present in 17/87 (19.5%) of probands with MRKHS and included seven deletions, three duplications, one new translocation in 5/50 cells-t(7;14)(q32;q32), confirmation of a previously identified translocation-t(3;16)(p22.3;p13.3), and two aneuploidies	Optical genome mapping was performed to identify Structural variants (SVs)
4.	Ma, C 2022 [59]	Cohort	622 probands with MRKHS	16 rare variants in TBX6 from the combined cohort, including 1 protein-truncating variant suggesting a causal role for 1. 7 were shown to induce a loss-of-function effect by impaired the normal splicing of TBX6 mRNA, decreased protein expression, perturbed transcriptional activity, and protein mislocalization.	
5.	Buchert, R, 2022 [53]	Case series	five MRKHS-discordant pairs of MZ twins<br>-1 had type-1	- mosaic variant in ACTR3B with a high allele frequency in the affected tissue, a low allele frequency in the blood of the affected twin and almost	Genome sequencing of blood of both twins as well as transcriptome

			-4 had type-2 MRKHS	absent in the blood of the unaffected twin- ACTR3B- Pathogenic<br>-PAX8 variant- a variant of unknown significance<br>-missense variant in WNT9B- a variant of unknown significance<br>-detected a pathogenic variant in *GREB1L* in one twin pair and their unaffected mother (showing a reduced phenotypic penetrance)<br>-transcriptome- widespread perturbations largely similar to those in sporadic cases. -Transcriptional changes were enriched for terms associated with estrogen and its receptors	analysis of uterine tissue of the affected twin
6.	Li, H 2022 [86]	Case control	40 MRKHS and 140 individuals controls	Four novel variations of EMX2	
7.	Chu, C, 2022 [44]	Case series	10 MRKHS individuals	Variants of nine genes: TBC1D1, KMT2D, HOXD3, DLG5, GLI3, HIRA, GATA3, LIFR, and CLIP1 (n = 9)	
8.	Dell'Edera D,2021 [158]	Case report	1	Microduplications in 22q11.21	
9.	Chen N, 2021 [38]	Case control	592 MRKHS individuals (442 Chinese and 150 of mixed ethnicity) 941 individual controls	Variants of 7 genes: PAX8 (n = 4), BMP4 (n = 2), BMP7 (n = 2), TBX6 (n = 1), HOXA10 (n = 1), EMX2 (n = 1), and WNT9B (n = 1)	Included patients had unilateral renal aplasia/ectopic kidney, and cervicothoracic somite dysplasia association
10.	Mikhael S, 2021 [39]	Cohort	111 MRKHS individuals type 1 (*n* = 82) type-2 (*n* = 29)	Variants of: WNT4, LAMC1, RARA, HOXA10, PAX2, and WNT9B, TBX6, SHOX, MMP14, and LRP10	
11.	Pontecorvi P,2021[45]	Cohort	36 MRKHS individuals	Altered gene expression pattern in PRKX, MUC1, HOXC8, GREB1L	
12.	Hentrich T, 2020 [97]	Case control	39 with MRKHS<br>type 1- 22<br>type 2- 17<br>30 patients with healthy endometrium	1906 differentially expressed genes (DEGs) comprising 1236 up and 670 downregulated genes in MRKHS type 1 and 1174 DEGs with 801 up- and 373 downregulated genes in MRKHS type 2 were identified when compared to controls	Also compared endometrial tissues. RNA-seq of endometrial tissue of uterus rudiments for transcriptome analysis

				-Gene expression changes during the menstrual cycle are missing in the endometrium of MRKHS patients	
13.	Jacquinet A,2020 [80]	Cohort	9 families with CUAs and/or kidney malformations 68 individuals with CUAs	Variants of GREB1L (n = 4 families and 5 individuals)	Also included family members with only kidney malformations
14.	Anant M, 2020 [154]	Case report	1 with MRKHS type 2	18p deletion (n = 1)	
15.	Smol T, 2020 [88]	Case report	1 with MRKHS	Microdeletion in 2q12.1q14.1 (involving PAX8) and microdeletion of SHOX locus	Patient also had congenital hypothyroidism
16.	Backhouse B, 2019 [43]	Cohort	8 MRKHS patients<br>-6 had type-1<br>-2 had type-2	-Microarray analysis identified a 0.6-Mb deletion in the 16p11.2 region in a patient with MRKHS type 2 (location 29,595,483–30,199,713), affected candidate gene TBX6<br>- Variants (n = 6) and a deletion (affecting TBX6) (n = 1) of 16p11.2 8 MRKHS and MURCS individuals<br>-16 rare nonsynonymous variants in MRKHS candidate genes across the cohort, including variants in several genes, such as LRP10 and DOCK4	whole exome sequencing was used
17.	Herlin M K, 2019 [79]	Case series	4 (Three genera-tions family)	Variants of GREB1L	
18.	Pan H X, 2019 [41]	Case series	9 MRKHS type 1 and their parents	De novo changes in BAZ2B, KLHL18, PIK3CD, SLC4A10 and TNK2	
19.	Tewes A C, 2019 [58]	Case control	26 MRKHS type 1<br>27 MRKHS type 2<br>135 individual controls	Variants and substitution of TBX6 (n = 4)	72 individuals with Müllerian duct fusion anomalies were also included
20.	Takahashi K, 2018 [95]	Case control	10 MRKHS, and 7 unaffected individuals	De novo variants of MYCBP2, NAV3, and PTPN3 (n = 3 families) and a variant of MYCBP2 (n = 1)	included three MRKHS persons from trio-based families
21.	Ledig S, 2018 [63]	Cohort	103 individuals with CUAs	Microdeletions and microduplications in 17q12, 22q11.21, 9q33.1, 3q26.11 and 7q31.1. (n = 8)	

22.	AlSubaihin A, 2018 [74]	Case report	1	Tetrasomy of the pericentromeric region of chromosome 22 (n = 1)	The patient had CES with MRKHS
23.	Eggermann T, 2018 [168]	Case series	MRKHS Type 1- 53 Type 2-52	MRKHS due to an ICR1 hypomethylation in 11p15.5. Failing to identify altered imprinting marks of differentially methylated regions PLAGL1, GRB10 and MEST, H19 and KCNQ1OT1, MEG3, SNRPN, DIRAS, NESPAS and GNAS. Abnormality present (n=1) absent (n=100)	Also included individuals with Silver Russel Syndrome
24.	Eksi D, 2018 [57]	Cohort	19 MRKHS individuals	Variants of BM8A, CMTM7, CCR4, TRIM71, CNOT10, TP63, EMX2, and CFTR (n = 4)	
25.	Zhang W, 2017 [68]	Case report	1 MRKHS	Novel missense mutation in LHX1 (NM_005568: c.G1108A, p.A370T)	Used whole-exome sequencing analysis
26.	Williams L S, 2017 [66]	Cohort	147 MRKHS from North America and Turkey	Copy number variants of WNT4, HNF1B, or LHX1 (n = 6), but no point change (n = 100)	Included MRKHS indivi-duals with family members affected, and singletons
27.	Brucker SY 2017 [83]	Cohort	93 MRKHS; 68 type 1 25 type 2	Variants of OXTR (n = 18) and ESR1 (n = 1)	
28.	Xing Q, 2016 [81]	Case control	200 individual controls	Missense change of DACT1 (n = 1)	Also included 100 individuals with other Müllerian duct anomalies
29.	Williams 2016 [40]	Case report	1 individual with MRKHSS	A balanced chromosomal translocation involving chromosomes 3 and 16	
30.	Waschk D E J, 2016 [54]	Case control	109 MRKHS and 135 individual controls	Variant of WNT9B (n = 5)	Also included individuals with Müllerian duct anomalies
31.	McGowan R, 2015 [48]	Prospe ctive study	11 with MRKHS	Microdeletion and microduplication 1q21.1, 7p14.3, 16p11.2, 17q12, and 22q11.21-q11.23 and possibly implicating several genes (LHX1, BBS9, HNF1b, and TBX6) (n = 9)	Included 24 individuals with other Müllerian disorders
32.	Ma,W. 2015 [51]	Case control	182 MRKHS; 155 type 1, 27 type 2 and 228 controls	Polymorphisms in WNT9B and PBX1; Epistatic effect of AMH, PBX1, WNT7A and WNT9B	All individuals were unrelated
33.	Chen M J, 2015 [23]	Cohort	7 MRKHS type 1 individuals	Deletions at 15q11.2 (80%), 19q13.31 (40%), 1p36.21 (40%) and 1q44 (40%)	

				(n = 5),1q21.1 (n = 2). Damaging variants of HNRNPCL1, OR2T2, OR4M2, ZNF816 and PDE11A	
34.	Rall, K. 2015 [25]	Cohort	5 MRKHS-discor -dant monozygotic twin pairs	Duplication of MMP14 and LRP10 (n = 1) affected twin	
35.	Liu S, 2015 [87]	Case control	517 cases and 563 controls	Novel nonsense variants of EMX2 (n = 1)	Included individuals with incomplete Müllerian Fusion
36.	Tewes A C, 2015 [50]	Retrospective case control	116 MRKHS and 94 individual control	Variants of RBM8A (n = 13) TBX6 (n = 5)	Also included 51 individuals with other MD abnormalities
37.	Murry, 2015 [99]	Cohort	20 individuals with CUA	No pathogenic Copy number changes (n = 20)	
38.	Wang M, 2014 [52]	Case control	42 MRKHS and 42 individual controls	Variants of WNT9B (n = 1)	
39.	Herlin. 2014 [19]	Case report	2 cousins with MRKHS	Familial occurrence of MRKHS and unilateral renal aplasia. Male cousins have unilateral renal aplasia.	Reported 67 familial cases of MRKHS with other associated anomalies
40.	Nodale C, 2014 [46]	Case control	16 MRKHS and 5 individual controls	Upregulation of MUC1 (n = 8) and significant upregulation of HOXC8 (n = 3). Downregulation of HOXB2 (n = 7) and HOXB5 (n = 7) and Notch ligands JAG1 (n = 6) and DLL1 (n = 5)	
41.	Ma, D. 2014 [22]	Case report	1 with Müllerian agenesis and hypothyroidism	Deletion at 2q13q14.2 (including PAX8) (n = 1)	
42.	Sandbacka M,2013 [56]	Case control	112 MRKHS I and 200 individual control	Variations including 16p11.2 and 17q12 deletions (8/50) or variations in TBX6 or LHX1 in MA patients (30/112)	Controls were women with at least 1 child
43.	Ekici AB, 2013 [142]	Case control	20 MRKHS and 53 individual control	Variations HOXA10 and HOXA13	Included 7 non-MRKHS individuals with genital tract anomalies
44.	Wang P, 2012 [100]	Case control	15 with uterine aplasia and 192	Variant of PAX2 (n = 1)	Included 177 with

			ethnic-matched individual controls		incomplete Müllerian fusion
45.	Ledig S, 2012 [61]	Cohort	23 MRKHS I<br>39 MRKHS II	No changes in HNF1B Variants of LHX1 (n = 1/62)	Included 2 patients with DM and learning disability
46.	Xia M, 2012 [67]	Case control	96 with CUAs and 105 individual controls	No significant variants (n = 0/96) but a rare polymorphism of LHX1 (n = 1/77)	Looked for variants of LHX
47.	Hinkes B, 2012 [62]	Case report	1 with MRKHS	Microdeletion in 17q12 (involving HNF1b and LHX1) (n = 1)	The patient also had unilateral renal aplasia
48.	Chang X, 2012 [101]	Case series	10 MRKHS	No perturbation that indicates the significance of WNT4	Included 5 subjects with Müllerian aplasia and 174 incomplete Müllerian fusion
49.	Ravel C, 2012 [82]	Case control	12 MRKHS individuals	No significant changes were observed between the MRKHS individuals and the control group for LAMC1 and DLGH1 gene polymorphisms.	
50.	Philibert P 2011[75]	Cohort	4 MRKHS	Wnt4 mutation	Included those with Mullerian duct abn. and hyperandrogenism
51.	Ledig S, 2011 [21]	Cohort	56 MRKHS individuals	Microdeletions and -duplications in 1q21.1, 17q12, and 22q11.21 involving LHX1 and HNF1B gene (n = 48)	
52.	Ma J, 2011 [90]	Cohort	192 Chinese individuals with CUAs	Polymorphisms in PBX1 (n = 2)	
53.	Sandbacka M, 2011 [167]	Cohort	83 individuals with CUAs	No association between hypomethylation of the H19 imprinted control region but aberrant methylation (n = 3/16)	
54.	Nik-Zainal S, 2011[55]	Cohort	38 MRKHS I<br>25 MRKHS II	Microdeletion at 16p11.2 (n = 4), microdeletion at 17q12 (n = 4), 22q11.2 (n = 1)	Included isolated and syndromic MA
55.	Rall K, 2011 [32]	Case control	8 MRKHS and 8 individual controls	293 genes with altered expression and 194 genes differentially methylated.	
56.	Morcel K, 2011 [72]	Cohort	57 MRKHS individuals	Deletion in 4q34-qter, 8p23.1, 10p14 and 22q11.2 (n = 4)	Included individuals with

					DiGeorge syndrome and other multiple abnormalities
57.	Gervasini C, 2010 [89]	Case control	30 MRKHS and 53 individual controls	Partial duplication of SHOX (n = 5)	
58.	Acién P, 2010 [105]	Case report	1 MRKHS	No microdeletions in 17q12 and 22q11.21 (n = 1)	The patient also had pulmonary hypoplasia
59.	Oram RA, 2010 [69]	Cohort	58 individuals with isolated CUAs	Variants or deletion of HNF1B (n = 9/50 individuals with both CUAs and renal abnormalities)	Included 50 individuals with both CUAs and renal abnormalities
60.	Liatsikos S A, 2010 [102]	Case control	30 with MDAs and 100 individual controls	No causative variants of HOX A10 and HOX A11	
61.	Ravel C, 2009 [145]	Cohort	11 MRKHS individuals	Variants of WNT4, WNT5A, WNT7A, and WNT9B	
62.	Bernardini L,2009 [60]	Case series	22 MRKHS individuals	Deletion in 17q12 (involving TCF2 and LHX1 genes) (n = 2)	
63.	Hofstetter G,2008 [103]	Case report	1 MURCS	No major deletions or duplications in 22q11.1 12q24.1. and 3q27 (n = 1)	
64.	Lalwani S, 2008 [94]	Case control	26 individuals with CUAs 30 controls	No HOXA10 gene variants	
65.	Philibert P, 2008 [144]	Case control	28 individuals with CUAs and 100 controls	Variants of WNT4 gene	
66.	Mencarelli M A, 2008 [64]	Case series	84 with mental problems and Uterine aplasia	Deletions in 7q31, 14q21.1, Xq25 and duplications in 12p11.22, 12q21.31, 13q31.1, 17q12, Xp22.31, Xq28 (n=10 CNVs). Parents were healthy	Primarily included individuals with mental problems
67.	Miyamoto 2008 [85]		Human and other animal models	Showed GATA4 a key regulator of gonadal development by regulating SRY and AMH	
68.	Drummond JB, 2008 [93]	Cohort	12 MRKHS patients	No variants of the GSK-3beta phosphorylation sites on exon 3 of beta-catenin gene (n = 12)	

69.	Sundaram U T, 2007 [71]	Case report	2 with absent uterus	Deletion in 22q11.2 (n = 2)	Both patients had MA and unilateral renal agenesis
70.	Biason-Lauber A, 2007 [143]	Case report	1 with MRKHS	Variants of WNT4 (n = 1)	
71.	Cheroki C, 2007 [47]	Cohort	14 MRKHS II	Submicroscopic genomic imbalances in 1q21.1, 17q12, 22q11.21, and Xq21.31 (n=4)	
72.	Cheroki C, 2006 [73]	Cohort	25 MRKHS individuals	Deletion in 22q11 (excluding WNT-4, RARgamma, RXR-alpha) (n = 1)	
73.	Burel A, 2006 [104]	Cohort	6 MRKHS individuals	No variants of HOXA7-HOXA13 region (n = 6)	
74.	Oppelt P, 2005 [17]	Case control	30 MRKHS and 48 individual controls	AMH promoter sequence variations cannot be the cause of aberrant AMH expression leading to Müllerian duct formation disorders	
75.	Clément-Ziza Mi, 2005 [107]	Cohort	19 MRKHS individuals	No significant variations of WNT4 (n = 19)	
76.	Biason-Lauber A, 2004 [77]	Case report	1 with MRKHS	Variants of the WNT4 (n = 1)	
77.	Plevraki E, 2004 [110]	Case series	6 MRKHS individuals	Positive TSPY gene (n = 2)	
78.	Zenteno J C, 2004 [108]	Case control	15 with Mullerian agenesis and 25 individual controls	No significant difference in Polymorphisms AMH and AMHR genes between MRKHS individuals and controls	
79.	Klipstein S, 2003 [109]	Case control	32 with CUAs 138 controls	GALT enzyme do not affect PMD formation	
80.	Timmreck LS, 2003 [92]	Cohort	25 individuals with CUAs	Variants of CFTR (n = 2)	
81.	Aydos S, 2003 [161]	Case report	1	Deletion of Xq (n = 1)	MRKHS and gonadal dysgenesis
82.	Bingham C, 2002 [70]	Cohort	9 families	Changes in HNF-1beta gene (n = 2 families)	Included those with renal abnormalities and personal or family history of female genital tract malfor-mations,

					but no history of diabetes
83.	Resendes D L, 2001 [106]	Case control	22 with CUAs 96 individual controls	No changes or rare polymorphism in AMH and the AMHR genes (n = 22)	
84.	Lindner T H, 1999 [65]	Cohort	1 family	Deletion in HNF-1beta gene in 4 females. Mullerian aplasia (n=2)	Family with severe genital malformations progressive non-diabetic renal disease and mild DM
85.	Cramer DW, 1996 [111]	Case control	13 MRKHS and their mothers; 113 individual controls	Carriers for the N314D variants of GALT (n = 6/13 individuals with Müllerian agenesis and 16/113 individual controls)	Included cases with vaginal agenesis and rudimentary uterus

[CES= Cat Eye Syndrome, CUA= Complete Uterine Aplasia]

2. EXPANDED NAMES FOR GENES

- CMTM7= CKLF Like MARVEL Transmembrane Domain Containing 7
- MEFV= Familial Mediterranean fever
- IL-32= Interleukin 32
- BAZ2B = Bromodomain Adjacent To Zinc Finger Domain Protein 2B
- KLHL18= Kelch Like Family Member 18
- PIK3CD= Phosphatidylinositol-4,5-Bisphosphate 3-Kinase Catalytic Subunit Delta
- SLC4A10= Solute Carrier Family 4 Member 10
- TNK2= Tyrosine Kinase Non Receptor 2
- PAX= Paired-box gene
- LAMC1= Laminin Subunit Gamma 1
- RARA= Retinoic Acid Receptor Alpha
- HOXA10= Homeobox A10
- SHOX= Short stature Homeobox-containing gene
- MMP14= Matrix Metallopeptidase 14
- LRP10= LDL Receptor Related Protein 10
- IFTP57= Intraflagellar transport protein 57
- HHLA2= Human Endogenous Retrovirus-H Long Terminal Repeat-Associating Protein 2
- MYH15= Myosin Heavy chain 15
- PLA2R1= Phospholipase A2 Receptor 1
- ITGB6= Integrin Subunit Beta 6
- RBMS1= RNA Binding Motif Single Stranded Interacting Protein 1
- FRAS1= Fraser Extracellular Matrix Complex Subunit 1
- CC2D2A= Coiled-Coil And C2 Domain Containing 2A

- KIF14= Kinesin Family Member 14
- RSPO4= R-Spondin 4
- MKKS= McKusick-Kaufman/Bardet-Biedl Syndrome Centrosomal Shuttling Protein
- NPHP3= Nephrocystin 3
- DYNC2H1= Dynein Cytoplasmic 2 Heavy Chain 1
- DOCK4= Dedicator Of Cytokinesis 4
- SPECC1L= Sperm antigen with calponin homology and Coiled-Coil domains 1 Like
- VWF= Von Willebrand Factor
- TBC1D1= TBC1 Domain Family Member 1
- KMT2D= Lysine Methyltransferase 2D
- HOXD3= Homeobox D3
- DLG5= Discs Large MAGUK Scaffold Protein 5
- GLI3= Glioma-Associated Oncogene Family Zinc Finger 3
- HIRA= Histone Cell Cycle Regulator
- GATA= Globin Transcription Factor Binding Protein
- LIFR= Leukemia Inhibitory Factor Receptor Alpha
- CLIP1= CAP-Gly Domain Containing Linker Protein 1
- PRKX= Protein Kinase CAMP-Dependent X-Linked Catalytic Subunit
- MUC1= Mucin 1
- HOXC8= Homeobox C8
- RBM8A= RNA-binding protein 8A
- WNT9B= WNT Family Member 9B
- TBX= T-Box Transcription Factor
- TCF2= Transcription Factor 2; Also called HNF1B
- HNF1B= Hepatocyte Nuclear Factor 1 Homeobox B; Also called TCF2
- ACTR3B= Actin-related protein 3B
- LHX1= LIM Homeobox 1
- WNT= Wingless-related integration site
- GREB1L= Growth Regulation By Estrogen In Breast Cancer 1-Like Protein
- ZNF= Zinc finger protein
- DLGH1= Discs Large Homolog 1
- OXTR= Oxytocin Receptor
- ESR1= Estrogen Receptor 1
- WT1= Wilms Tumor 1
- EMX2= Empty Spiracles Homeobox 2
- WISP2= WNT1 inducible signaling pathway protein 2
- DACT1= Dishevelled Binding Antagonist Of Beta Catenin 1
- HOXA5= Homeobox A5
- HOXA9= Homeobox A9
- TRIM71= tripartite motif containing 71
- CCR4= C-C motif chemokine receptor 4
- CNOT10= CCR4-NOT transcription complex subunit 10
- OR1F1= Olfactory receptor family 1 subfamily F member 1
- PBX1= Pre-B-Cell Leukemia Homeobox 1
